# (2*S*,3*R*)-*tert*-Butyl *N*-[4-(*N*-benzyl-4-fluoro­benzene­sulfonamido)-3-hy­droxy-1-phenyl­butan-2-yl]carbamate

**DOI:** 10.1107/S1600536812011440

**Published:** 2012-03-21

**Authors:** Marcele Moreth, Marcus V.N. de Souza, James L. Wardell, Solange M. S. V. Wardell, Edward R. T. Tiekink

**Affiliations:** a Instituto de Tecnologia em Fármacos–Farmanguinhos, FioCruz–Fundação Oswaldo Cruz, R. Sizenando Nabuco, 100, Manguinhos, 21041-250 Rio de Janeiro, RJ, Brazil; b Centro de Desenvolvimento Tecnológico em Saúde (CDTS), Fundação Oswaldo Cruz (FIOCRUZ), Casa Amarela, Campus de Manguinhos, Av. Brasil 4365, 21040-900 Rio de Janeiro, RJ, Brazil; c CHEMSOL, 1 Harcourt Road, Aberdeen, AB15 5NY, Scotland; d Department of Chemistry, University of Malaya, 50603 Kuala Lumpur, Malaysia

## Abstract

In the title mol­ecule, C_28_H_33_FN_2_O_5_S, the mean plane about the tertiary amine group (sum of the angles subtended at the *sp* ^2^-hybridized N atom = 359.7°) forms a dihedral angle of 16.66 (6)° with the phenyl ring adjacent to the carbamate group. The sulfonamide benzene ring and the hy­droxy group lie to either side of the C_2_NS plane, whereas the benzyl­phenyl (connected to the N atom) and carbamate substituents lie to the other side. Supra­molecular layers propagating in the *ac* plane are found in the crystal, linked by hy­droxy–sulfonamide O—H⋯O and carbamate–carbamate N—H⋯O hydrogen bonds along with C—H⋯O and C—H⋯π inter­actions.

## Related literature

For background to tuberculosis (TB) infection, see: de Souza (2006 ▶). For the development of β-amino­alcohols for the treatment of patients co-infected with TB and HIV, see: Yendapally & Lee (2008 ▶); Ferreira *et al.* (2009 ▶); Cunico *et al.* (2008 ▶, 2011 ▶); Gomes *et al.* (2011 ▶).
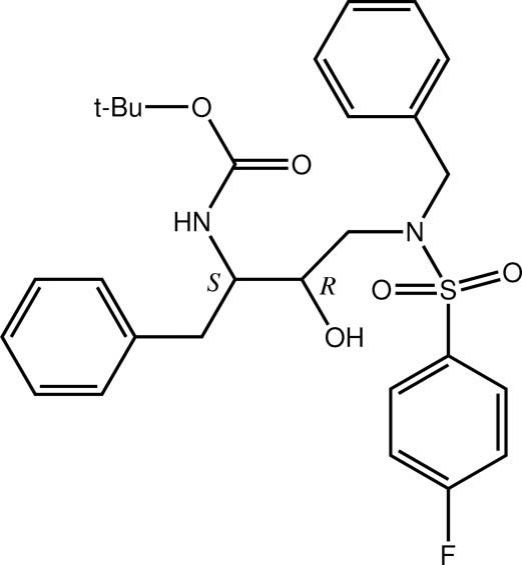


## Experimental

### 

#### Crystal data

C_28_H_33_FN_2_O_5_S
*M* *_r_* = 528.62
Monoclinic, 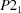

*a* = 5.4116 (1) Å
*b* = 25.5513 (8) Å
*c* = 9.8615 (3) Å
β = 101.641 (2)°
*V* = 1335.54 (6) Å^3^
*Z* = 2
Mo *K*α radiation
μ = 0.17 mm^−1^
*T* = 120 K
0.50 × 0.24 × 0.18 mm

#### Data collection

Bruker–Nonius Roper CCD camera on a κ-goniostat diffractometer
Absorption correction: multi-scan (*SADABS*; Sheldrick, 2007 ▶) *T*_min_ = 0.654, *T*_max_ = 0.746
12832 measured reflections
5956 independent reflections
5075 reflections with *I* > 2σ(*I*)
*R*_int_ = 0.040

#### Refinement

*R*[*F* ^2^ > 2σ(*F* ^2^)] = 0.042
*wR*(*F* ^2^) = 0.103
*S* = 1.01
5956 reflections
343 parameters
3 restraints
H atoms treated by a mixture of independent and constrained refinement
Δρ_max_ = 0.21 e Å^−3^
Δρ_min_ = −0.33 e Å^−3^
Absolute structure: Flack (1983 ▶), 2848 Friedel pairs
Flack parameter: 0.06 (6)

### 

Data collection: *COLLECT* (Hooft, 1998 ▶); cell refinement: *DENZO* (Otwinowski & Minor, 1997 ▶) and *COLLECT*; data reduction: *DENZO* and *COLLECT*; program(s) used to solve structure: *SHELXS97* (Sheldrick, 2008 ▶); program(s) used to refine structure: *SHELXL97* (Sheldrick, 2008 ▶); molecular graphics: *ORTEP-3* (Farrugia, 1997 ▶) and *DIAMOND* (Brandenburg, 2006 ▶); software used to prepare material for publication: *publCIF* (Westrip, 2010 ▶).

## Supplementary Material

Crystal structure: contains datablock(s) global, I. DOI: 10.1107/S1600536812011440/hb6679sup1.cif

Structure factors: contains datablock(s) I. DOI: 10.1107/S1600536812011440/hb6679Isup2.hkl

Supplementary material file. DOI: 10.1107/S1600536812011440/hb6679Isup3.cml

Additional supplementary materials:  crystallographic information; 3D view; checkCIF report

## Figures and Tables

**Table 1:** Hydrogen-bond geometry (Å, °) *Cg*1 is the centroid of the C23–C28 ring.

*D*—H⋯*A*	*D*—H	H⋯*A*	*D*⋯*A*	*D*—H⋯*A*
N2—H2n⋯O4^i^	0.88 (2)	2.36 (2)	3.179 (2)	154 (2)
O3—H3o⋯O2^i^	0.84 (2)	2.08 (2)	2.904 (2)	166 (3)
C7—H7*A*⋯O2^i^	0.99	2.56	3.405 (3)	143
C22—H22*A*⋯O4^i^	0.99	2.57	3.358 (2)	137
C20—H20*C*⋯*Cg*1^ii^	0.98	2.78	3.719 (2)	160

Symmetry codes: (i) 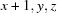; (ii) 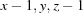.

**Fig. 1.**
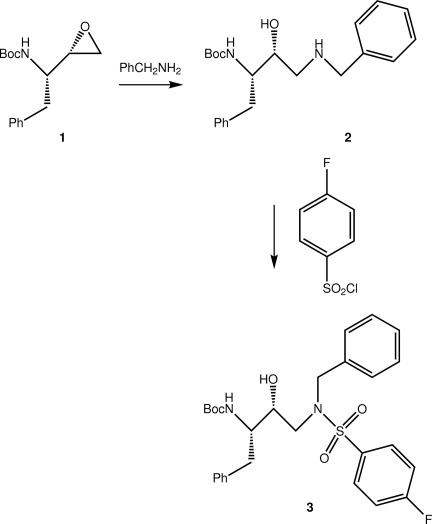
Reaction scheme for the synthesis of the title compound, 3.

**Fig. 2.**
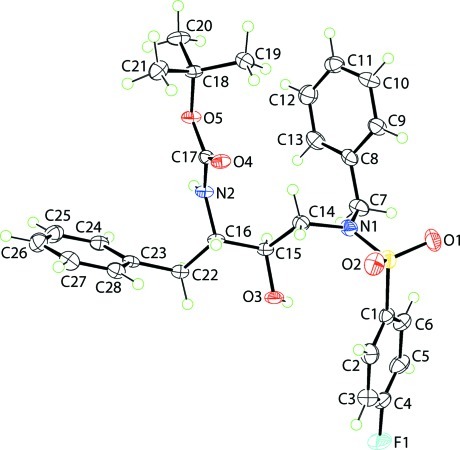
The molecular structure of 3 showing displacement ellipsoids at the 50% probability level.

**Fig. 3.**
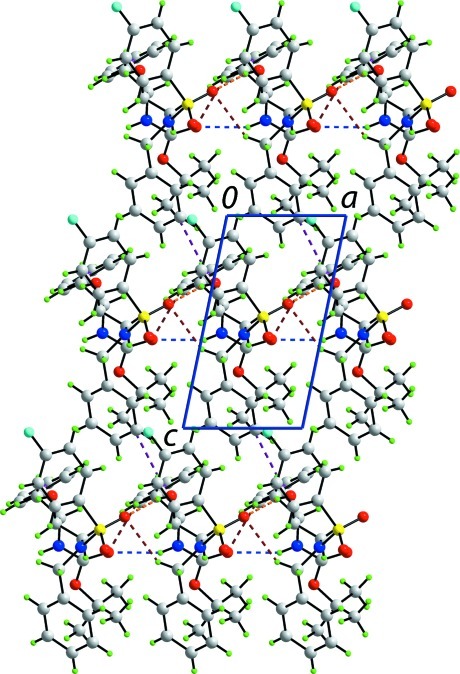
A view of the supramolecular layer in in the ac plane (I) in 3. The O—H···O, N—H···O, C—H···O and C—H···π interactions are shown as orange, blue, brown and purple dashed lines, respectively.

**Fig. 4.**
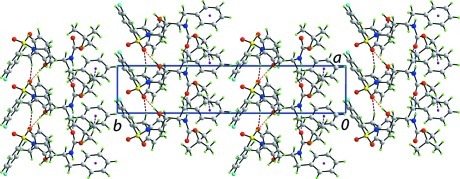
A view in projection down the c axis of the stacking of supramolecular layers in 3 along the b axis. The O—H···O, N—H···O, C—H···O and C—H···π interactions are shown as orange, blue, brown and purple dashed lines, respectively.

## supplementary crystallographic information

### Comment

Tuberculosis, TB, once again is becoming a worldwide problem due mainly to the increase in numbers of people infected with the HIV virus (de Souza, 2006). In 2010, there were 1.1 million new cases of TB among people living with HIV. Of the 1.8 million HIV-related deaths in 2010, 350,000 were due to TB (www.who.int/hiv/topics/tb/hiv_tb_factsheet_june_2011). Beta-aminoalcohols are actively being considered as a promising class of compounds in the combat of co-infection by TB and HIV, due to its versatility and significant application in anti-TB and anti-HIV treatment (Yendapally *et al.*, 2008; Ferreira *et al.*, 2009; Cunico *et al.*, 2008; Cunico *et al.*, 2011; Gomes *et al.*, 2011). We now wish to report the structure of the title compound, 3, prepared as shown in Fig. 1.

Compound 3, Fig. 2, the tertiary amine is *sp*^2^ hybridized as evidenced by the sum of the angles subtended at the N1 atom of 359.7°. The sulfonamide-benzene and benzyl-phenyl rings connected to the N1 atom are almost orthogonal to each other, with the dihedral angle between them being 73.86 (11)°, and lie to either side of the C_2_NS plane. The hydroxy group is orientated to the same side of the molecule as the sulfonamide-benzene ring, and the carbamate group to the same side as the benzyl-phenyl ring. The dihedral angle between the carbamate group and adjacent phenyl ring is 66.35 (8)°, with the latter approximately parallel with the C_2_NS plane, forming a dihedral angle of 16.66 (6)°.

In the crystal packing, the hydroxy group forms a hydrogen bond with a sulfonamide-O atom, and the carbamate-N—H and O atoms self-associate, Table 1. These interactions are reinforced by C—H···O and C—H···π interactions, Table 1, to form a supramolecular layer in the *ac* plane, Fig. 3. Layers stack along the *b* axis with no specific intermolecular interactions between them, Fig. 4.

### Experimental

A solution of (2*S*,3*S*)-*boc*-phenylalanine epoxide, 1, (1.6 mmol) and benzylamine (1.5 mmol) in i-PrOH (10 ml) was refluxed for 16 h. The reaction mixture was rotary evaporated and the crude product, 2, was crystallized from aqueous MeOH. To a solution of 2 in CH_2_Cl_2_ (10 ml) was successively added trifluoroacetic acid (2.2 mmol) and DMF (0.2 mmol). The mixture was stirred for 30 min under nitrogen, 4-fluorobenzenesulfonyl chloride (2.0 mmol) was added portion wise, After stirring for 8 h, the reaction mixture was washed with 5% HCl aqueous solution, water, brine, dried over MgSO_4_ and rotary evaporated to give the title compound, 3, which was recrystallized from EtOH to provide colourless blocks, *M*.pt: 418–420 K.

^1^H NMR δ: 7.92–7.88 (m, 2H, Ph); 7.43–7.38 (m, 2H, Ph); 7.32–7.25 (m, 5H, Ph); 7.23–7.19 (m, 2H, Ph); 7.15–7.11 (m, 3H, Ph); 6.60 (d, 1H, *J*_H,H_ = 8.8, NH); 4.97 (d, 1H, *J*_H,H_ = 6.0, OH); 4.57 (d, 1H, *J*_H,H_ = 15.6; H5a); 4,41 (d, 1H, *J*_H,H_ = 15.6, H5b); 3.51–3.45 (m, 2H, H3 and H2); 3.38–3.32 (m, 1H, H4a); 3.00 (dd, 1H, *J*_H,H_ = 14.8, ^2^*J*_H,H_ = 8.8, H4b); 2.90 (dd, 1H, *J*_H,H_ = 14.4, ^2^*J*_H,H_ = 2.8, H1a); 2.45 (dd, 1H, *J*_H,H_ = 13.6, ^2^*J*_H,H_ = 10.4, H1b); 1.21 (s, 9H, C(CH_3_)_3_). ^13^C NMR δ: 164.2 (d, *J*_C,F_= 254.6, C4'); 155.2 (C═ O); 139.4; 136.6 (d, ^4^*J*_C,F_ = 2.6, C1'); 136.5; 129.9 (d, ^3^*J*_C,F_ = 9.4, C2' and C6'); 128.2, 128.0, 127.8, 116.2 (d, ^2^*J*_C,F_ = 22.4, C3' and C5'); 77.4 (C(CH_3_)_3_); 71.7 (C3); 54.9 (C2); 51.2 (C5); 50.4 (C4); 35.2 (C1); 28.1 (C(CH_3_)_3_).

### Refinement

The C-bound H atoms were geometrically placed (C—H = 0.95–1.00 Å) and refined as riding with *U*_iso_(H) = 1.2–1.5*U*_eq_(C). The O-bound H-atom was located in a difference Fourier map and refined with an O—H restraint of 0.84±0.01 Å, and with *U*_iso_(H) = 1.5*U*_eq_(O); the N—H H-atom was treated similarly with N—H = 0.88±0.01 Å and with *U*_iso_(H) = 1.2*U*_eq_(N).

### Crystal data

**Table:**

C_28_H_33_FN_2_O_5_S	*F*(000) = 560
*M**_r_* = 528.62	*D*_x_ = 1.315 Mg m^−^^3^
Monoclinic, *P*2_1_	Mo *K*α radiation, λ = 0.71073 Å
Hall symbol: P 2yb	Cell parameters from 11534 reflections
*a* = 5.4116 (1) Å	θ = 2.9–27.5°
*b* = 25.5513 (8) Å	µ = 0.17 mm^−^^1^
*c* = 9.8615 (3) Å	*T* = 120 K
β = 101.641 (2)°	Block, colourless
*V* = 1335.54 (6) Å^3^	0.50 × 0.24 × 0.18 mm
*Z* = 2	

### Data collection

**Table:**

Bruker–Nonius Roper CCD camera on a κ-goniostat diffractometer	5956 independent reflections
Radiation source: Bruker–Nonius FR591 rotating anode	5075 reflections with *I* > 2σ(*I*)
Graphite monochromator	*R*_int_ = 0.040
Detector resolution: 9.091 pixels mm^-1^	θ_max_ = 27.5°, θ_min_ = 3.2°
φ and ω scans	*h* = −7→6
Absorption correction: multi-scan (*SADABS*; Sheldrick, 2007)	*k* = −32→33
*T*_min_ = 0.654, *T*_max_ = 0.746	*l* = −12→12
12832 measured reflections	

### Refinement

**Table:**

Refinement on *F*^2^	Secondary atom site location: difference Fourier map
Least-squares matrix: full	Hydrogen site location: inferred from neighbouring sites
*R*[*F*^2^ > 2σ(*F*^2^)] = 0.042	H atoms treated by a mixture of independent and constrained refinement
*wR*(*F*^2^) = 0.103	*w* = 1/[σ^2^(*F*_o_^2^) + (0.0535*P*)^2^] where *P* = (*F*_o_^2^ + 2*F*_c_^2^)/3
*S* = 1.01	(Δ/σ)_max_ < 0.001
5956 reflections	Δρ_max_ = 0.21 e Å^−^^3^
343 parameters	Δρ_min_ = −0.33 e Å^−^^3^
3 restraints	Absolute structure: Flack (1983), 2848 Friedel pairs
Primary atom site location: structure-invariant direct methods	Flack parameter: 0.06 (6)

### Special details

**Table:**

Geometry. All s.u.'s (except the s.u. in the dihedral angle between two l.s. planes) are estimated using the full covariance matrix. The cell s.u.'s are taken into account individually in the estimation of s.u.'s in distances, angles and torsion angles; correlations between s.u.'s in cell parameters are only used when they are defined by crystal symmetry. An approximate (isotropic) treatment of cell s.u.'s is used for estimating s.u.'s involving l.s. planes.
Refinement. Refinement of *F*^2^ against ALL reflections. The weighted *R*-factor *wR* and goodness of fit *S* are based on *F*^2^, conventional *R*-factors *R* are based on *F*, with *F* set to zero for negative *F*^2^. The threshold expression of *F*^2^ > 2σ(*F*^2^) is used only for calculating *R*-factors(gt) *etc*. and is not relevant to the choice of reflections for refinement. *R*-factors based on *F*^2^ are statistically about twice as large as those based on *F*, and *R*- factors based on ALL data will be even larger.

### Fractional atomic coordinates and isotropic or equivalent isotropic displacement parameters (Å^2^)

**Table:**

	*x*	*y*	*z*	*U*_iso_*/*U*_eq_	
S1	0.52180 (9)	0.91034 (2)	0.52242 (6)	0.02540 (14)	
F1	1.2856 (3)	0.99049 (6)	0.98111 (16)	0.0480 (4)	
O1	0.4439 (3)	0.95265 (6)	0.42835 (19)	0.0382 (4)	
O2	0.3377 (3)	0.88381 (6)	0.58349 (18)	0.0320 (4)	
O3	0.9825 (3)	0.81562 (6)	0.68221 (15)	0.0258 (3)	
H3O	1.088 (4)	0.8377 (8)	0.667 (3)	0.039*	
O4	0.4133 (2)	0.69554 (6)	0.41684 (14)	0.0213 (3)	
O5	0.6340 (3)	0.65982 (6)	0.26540 (15)	0.0221 (3)	
N1	0.6512 (3)	0.86650 (7)	0.44103 (19)	0.0247 (4)	
N2	0.8407 (3)	0.70276 (7)	0.44759 (18)	0.0196 (4)	
H2N	0.976 (3)	0.6946 (9)	0.415 (2)	0.024*	
C1	0.7491 (4)	0.93595 (8)	0.6604 (2)	0.0227 (5)	
C2	0.7357 (4)	0.92322 (9)	0.7956 (3)	0.0305 (5)	
H2	0.6034	0.9015	0.8134	0.037*	
C3	0.9165 (5)	0.94237 (10)	0.9048 (3)	0.0369 (6)	
H3	0.9092	0.9344	0.9980	0.044*	
C4	1.1047 (5)	0.97287 (9)	0.8743 (3)	0.0335 (6)	
C5	1.1211 (4)	0.98673 (10)	0.7422 (3)	0.0333 (6)	
H5	1.2540	1.0086	0.7260	0.040*	
C6	0.9407 (4)	0.96826 (9)	0.6328 (3)	0.0274 (5)	
H6	0.9473	0.9775	0.5402	0.033*	
C7	0.7982 (5)	0.88253 (9)	0.3363 (2)	0.0286 (5)	
H7A	0.9790	0.8747	0.3714	0.034*	
H7B	0.7809	0.9208	0.3209	0.034*	
C8	0.7080 (4)	0.85424 (8)	0.2005 (2)	0.0237 (5)	
C9	0.4706 (4)	0.86529 (9)	0.1202 (2)	0.0286 (5)	
H9	0.3643	0.8901	0.1520	0.034*	
C10	0.3890 (4)	0.84028 (10)	−0.0058 (2)	0.0294 (5)	
H10	0.2262	0.8477	−0.0593	0.035*	
C11	0.5432 (4)	0.80475 (9)	−0.0538 (2)	0.0299 (5)	
H11	0.4876	0.7881	−0.1407	0.036*	
C12	0.7775 (5)	0.79355 (10)	0.0244 (3)	0.0353 (6)	
H12	0.8841	0.7690	−0.0081	0.042*	
C13	0.8579 (4)	0.81823 (10)	0.1514 (2)	0.0311 (5)	
H13	1.0195	0.8101	0.2053	0.037*	
C14	0.6509 (4)	0.81084 (8)	0.4777 (2)	0.0227 (5)	
H14A	0.5950	0.7903	0.3919	0.027*	
H14B	0.5254	0.8054	0.5368	0.027*	
C15	0.9042 (4)	0.78940 (8)	0.5533 (2)	0.0188 (4)	
H15	1.0331	0.7949	0.4949	0.023*	
C16	0.8788 (4)	0.73051 (8)	0.5795 (2)	0.0185 (4)	
H16	0.7262	0.7251	0.6205	0.022*	
C17	0.6120 (4)	0.68657 (8)	0.3804 (2)	0.0184 (4)	
C18	0.4078 (4)	0.64183 (8)	0.1668 (2)	0.0216 (5)	
C19	0.2370 (4)	0.68815 (9)	0.1142 (2)	0.0282 (5)	
H19A	0.3397	0.7173	0.0918	0.042*	
H19B	0.1466	0.6992	0.1860	0.042*	
H19C	0.1152	0.6778	0.0309	0.042*	
C20	0.5227 (4)	0.61986 (10)	0.0506 (2)	0.0308 (5)	
H20A	0.6410	0.5918	0.0868	0.046*	
H20B	0.6125	0.6477	0.0123	0.046*	
H20C	0.3886	0.6059	−0.0223	0.046*	
C21	0.2728 (4)	0.59978 (9)	0.2324 (3)	0.0297 (5)	
H21A	0.1835	0.6159	0.2989	0.045*	
H21B	0.3963	0.5745	0.2804	0.045*	
H21C	0.1514	0.5818	0.1603	0.045*	
C22	1.1081 (4)	0.70785 (8)	0.6797 (2)	0.0207 (4)	
H22A	1.2620	0.7146	0.6427	0.025*	
H22B	1.1273	0.7259	0.7700	0.025*	
C23	1.0838 (4)	0.64964 (8)	0.7015 (2)	0.0198 (4)	
C24	1.2408 (4)	0.61396 (9)	0.6537 (2)	0.0240 (5)	
H24	1.3646	0.6263	0.6055	0.029*	
C25	1.2194 (4)	0.56056 (10)	0.6754 (3)	0.0312 (5)	
H25	1.3294	0.5366	0.6435	0.037*	
C26	1.0372 (5)	0.54250 (9)	0.7437 (3)	0.0320 (6)	
H26	1.0212	0.5060	0.7582	0.038*	
C27	0.8783 (4)	0.57718 (9)	0.7909 (3)	0.0287 (5)	
H27	0.7521	0.5645	0.8370	0.034*	
C28	0.9026 (4)	0.63017 (9)	0.7710 (2)	0.0236 (5)	
H28	0.7944	0.6538	0.8052	0.028*	

### Atomic displacement parameters (Å^2^)

**Table:**

	*U*^11^	*U*^22^	*U*^33^	*U*^12^	*U*^13^	*U*^23^
S1	0.0209 (3)	0.0212 (3)	0.0315 (3)	−0.0010 (2)	−0.0008 (2)	0.0019 (2)
F1	0.0509 (9)	0.0460 (9)	0.0388 (9)	−0.0038 (8)	−0.0106 (7)	−0.0181 (7)
O1	0.0352 (10)	0.0244 (9)	0.0473 (11)	0.0013 (7)	−0.0099 (8)	0.0079 (8)
O2	0.0186 (8)	0.0303 (9)	0.0468 (11)	−0.0037 (6)	0.0056 (7)	−0.0027 (8)
O3	0.0302 (8)	0.0282 (9)	0.0167 (8)	−0.0058 (7)	−0.0009 (6)	−0.0049 (7)
O4	0.0173 (7)	0.0293 (8)	0.0178 (8)	−0.0033 (6)	0.0050 (6)	−0.0059 (6)
O5	0.0160 (7)	0.0316 (8)	0.0176 (8)	−0.0012 (6)	0.0012 (6)	−0.0076 (6)
N1	0.0297 (10)	0.0237 (10)	0.0206 (10)	−0.0041 (8)	0.0050 (8)	0.0039 (8)
N2	0.0165 (9)	0.0280 (10)	0.0140 (9)	−0.0019 (7)	0.0025 (7)	−0.0040 (7)
C1	0.0194 (10)	0.0211 (11)	0.0269 (13)	0.0018 (9)	0.0028 (9)	−0.0028 (9)
C2	0.0316 (12)	0.0276 (13)	0.0335 (14)	−0.0022 (10)	0.0091 (10)	0.0039 (10)
C3	0.0454 (15)	0.0358 (14)	0.0272 (14)	0.0024 (12)	0.0016 (11)	−0.0034 (11)
C4	0.0383 (14)	0.0243 (12)	0.0329 (14)	0.0041 (10)	−0.0044 (11)	−0.0102 (10)
C5	0.0262 (12)	0.0296 (13)	0.0436 (16)	−0.0063 (10)	0.0059 (11)	−0.0124 (11)
C6	0.0282 (12)	0.0237 (11)	0.0309 (13)	−0.0039 (9)	0.0073 (10)	−0.0052 (10)
C7	0.0288 (12)	0.0325 (13)	0.0235 (12)	−0.0097 (10)	0.0028 (10)	0.0057 (10)
C8	0.0231 (11)	0.0250 (11)	0.0221 (12)	−0.0057 (9)	0.0027 (9)	0.0073 (9)
C9	0.0288 (12)	0.0340 (13)	0.0222 (12)	0.0030 (10)	0.0034 (9)	0.0024 (10)
C10	0.0241 (11)	0.0432 (14)	0.0192 (12)	−0.0031 (10)	−0.0001 (9)	0.0014 (10)
C11	0.0362 (13)	0.0315 (13)	0.0223 (12)	−0.0071 (10)	0.0064 (10)	0.0000 (10)
C12	0.0348 (13)	0.0356 (14)	0.0365 (15)	0.0038 (11)	0.0094 (11)	−0.0026 (11)
C13	0.0225 (11)	0.0396 (14)	0.0300 (14)	0.0011 (11)	0.0024 (9)	0.0064 (11)
C14	0.0224 (10)	0.0229 (11)	0.0217 (11)	−0.0047 (9)	0.0018 (8)	0.0005 (9)
C15	0.0187 (10)	0.0230 (11)	0.0146 (10)	−0.0048 (8)	0.0028 (8)	−0.0019 (8)
C16	0.0185 (10)	0.0244 (10)	0.0126 (10)	−0.0033 (8)	0.0031 (8)	−0.0034 (8)
C17	0.0213 (10)	0.0187 (10)	0.0150 (10)	−0.0010 (8)	0.0033 (8)	0.0003 (8)
C18	0.0178 (10)	0.0270 (11)	0.0179 (11)	−0.0037 (9)	−0.0015 (8)	−0.0059 (9)
C19	0.0291 (12)	0.0306 (13)	0.0228 (12)	0.0025 (10)	0.0004 (9)	0.0007 (10)
C20	0.0281 (12)	0.0425 (14)	0.0212 (12)	−0.0018 (11)	0.0035 (9)	−0.0140 (11)
C21	0.0283 (12)	0.0263 (12)	0.0344 (14)	−0.0023 (10)	0.0063 (10)	−0.0048 (10)
C22	0.0188 (10)	0.0265 (11)	0.0164 (11)	−0.0017 (9)	0.0024 (8)	−0.0024 (9)
C23	0.0179 (10)	0.0273 (12)	0.0121 (10)	0.0003 (9)	−0.0015 (8)	−0.0015 (8)
C24	0.0221 (10)	0.0316 (12)	0.0183 (11)	−0.0004 (9)	0.0044 (9)	−0.0028 (9)
C25	0.0314 (13)	0.0309 (13)	0.0311 (14)	0.0052 (10)	0.0058 (11)	−0.0085 (10)
C26	0.0330 (13)	0.0256 (12)	0.0349 (15)	−0.0027 (10)	0.0007 (11)	−0.0016 (10)
C27	0.0248 (12)	0.0338 (13)	0.0275 (13)	−0.0020 (10)	0.0051 (10)	0.0028 (10)
C28	0.0210 (11)	0.0292 (12)	0.0202 (12)	0.0016 (9)	0.0030 (8)	0.0002 (9)

### Geometric parameters (Å, º)

**Table:**

S1—O1	1.4313 (17)	C12—C13	1.391 (3)
S1—O2	1.4341 (17)	C12—H12	0.9500
S1—N1	1.618 (2)	C13—H13	0.9500
S1—C1	1.765 (2)	C14—C15	1.524 (3)
F1—C4	1.362 (3)	C14—H14A	0.9900
O3—C15	1.424 (2)	C14—H14B	0.9900
O3—H3O	0.840 (10)	C15—C16	1.537 (3)
O4—C17	1.222 (2)	C15—H15	1.0000
O5—C17	1.350 (2)	C16—C22	1.535 (3)
O5—C18	1.475 (2)	C16—H16	1.0000
N1—C14	1.468 (3)	C18—C20	1.517 (3)
N1—C7	1.483 (3)	C18—C21	1.516 (3)
N2—C17	1.346 (3)	C18—C19	1.527 (3)
N2—C16	1.459 (3)	C19—H19A	0.9800
N2—H2N	0.877 (10)	C19—H19B	0.9800
C1—C2	1.389 (3)	C19—H19C	0.9800
C1—C6	1.394 (3)	C20—H20A	0.9800
C2—C3	1.390 (3)	C20—H20B	0.9800
C2—H2	0.9500	C20—H20C	0.9800
C3—C4	1.363 (4)	C21—H21A	0.9800
C3—H3	0.9500	C21—H21B	0.9800
C4—C5	1.371 (4)	C21—H21C	0.9800
C5—C6	1.383 (3)	C22—C23	1.512 (3)
C5—H5	0.9500	C22—H22A	0.9900
C6—H6	0.9500	C22—H22B	0.9900
C7—C8	1.514 (3)	C23—C24	1.392 (3)
C7—H7A	0.9900	C23—C28	1.397 (3)
C7—H7B	0.9900	C24—C25	1.390 (3)
C8—C13	1.377 (3)	C24—H24	0.9500
C8—C9	1.395 (3)	C25—C26	1.380 (4)
C9—C10	1.388 (3)	C25—H25	0.9500
C9—H9	0.9500	C26—C27	1.380 (3)
C10—C11	1.380 (3)	C26—H26	0.9500
C10—H10	0.9500	C27—C28	1.378 (3)
C11—C12	1.375 (3)	C27—H27	0.9500
C11—H11	0.9500	C28—H28	0.9500
			
O1—S1—O2	119.38 (10)	O3—C15—C16	109.35 (16)
O1—S1—N1	107.57 (11)	C14—C15—C16	109.39 (16)
O2—S1—N1	106.73 (10)	O3—C15—H15	109.3
O1—S1—C1	106.72 (10)	C14—C15—H15	109.3
O2—S1—C1	106.66 (10)	C16—C15—H15	109.3
N1—S1—C1	109.56 (10)	N2—C16—C22	109.95 (16)
C15—O3—H3O	104.1 (18)	N2—C16—C15	109.11 (16)
C17—O5—C18	120.63 (16)	C22—C16—C15	112.91 (16)
C14—N1—C7	117.84 (19)	N2—C16—H16	108.3
C14—N1—S1	121.78 (15)	C22—C16—H16	108.3
C7—N1—S1	120.07 (16)	C15—C16—H16	108.3
C17—N2—C16	122.62 (17)	O4—C17—N2	125.01 (19)
C17—N2—H2N	119.9 (15)	O4—C17—O5	125.03 (18)
C16—N2—H2N	117.3 (15)	N2—C17—O5	109.95 (17)
C2—C1—C6	120.7 (2)	O5—C18—C20	101.61 (16)
C2—C1—S1	119.32 (17)	O5—C18—C21	110.29 (17)
C6—C1—S1	119.95 (18)	C20—C18—C21	111.49 (18)
C3—C2—C1	119.8 (2)	O5—C18—C19	110.33 (17)
C3—C2—H2	120.1	C20—C18—C19	110.02 (19)
C1—C2—H2	120.1	C21—C18—C19	112.58 (18)
C4—C3—C2	118.1 (2)	C18—C19—H19A	109.5
C4—C3—H3	121.0	C18—C19—H19B	109.5
C2—C3—H3	121.0	H19A—C19—H19B	109.5
F1—C4—C3	118.1 (2)	C18—C19—H19C	109.5
F1—C4—C5	118.4 (2)	H19A—C19—H19C	109.5
C3—C4—C5	123.5 (2)	H19B—C19—H19C	109.5
C4—C5—C6	118.8 (2)	C18—C20—H20A	109.5
C4—C5—H5	120.6	C18—C20—H20B	109.5
C6—C5—H5	120.6	H20A—C20—H20B	109.5
C5—C6—C1	119.0 (2)	C18—C20—H20C	109.5
C5—C6—H6	120.5	H20A—C20—H20C	109.5
C1—C6—H6	120.5	H20B—C20—H20C	109.5
N1—C7—C8	111.22 (18)	C18—C21—H21A	109.5
N1—C7—H7A	109.4	C18—C21—H21B	109.5
C8—C7—H7A	109.4	H21A—C21—H21B	109.5
N1—C7—H7B	109.4	C18—C21—H21C	109.5
C8—C7—H7B	109.4	H21A—C21—H21C	109.5
H7A—C7—H7B	108.0	H21B—C21—H21C	109.5
C13—C8—C9	118.4 (2)	C23—C22—C16	112.36 (17)
C13—C8—C7	121.49 (19)	C23—C22—H22A	109.1
C9—C8—C7	120.1 (2)	C16—C22—H22A	109.1
C10—C9—C8	120.3 (2)	C23—C22—H22B	109.1
C10—C9—H9	119.8	C16—C22—H22B	109.1
C8—C9—H9	119.8	H22A—C22—H22B	107.9
C11—C10—C9	120.3 (2)	C24—C23—C28	118.0 (2)
C11—C10—H10	119.8	C24—C23—C22	121.33 (19)
C9—C10—H10	119.8	C28—C23—C22	120.64 (19)
C12—C11—C10	119.8 (2)	C25—C24—C23	121.0 (2)
C12—C11—H11	120.1	C25—C24—H24	119.5
C10—C11—H11	120.1	C23—C24—H24	119.5
C11—C12—C13	119.8 (2)	C26—C25—C24	119.6 (2)
C11—C12—H12	120.1	C26—C25—H25	120.2
C13—C12—H12	120.1	C24—C25—H25	120.2
C8—C13—C12	121.3 (2)	C25—C26—C27	120.3 (2)
C8—C13—H13	119.3	C25—C26—H26	119.8
C12—C13—H13	119.3	C27—C26—H26	119.8
N1—C14—C15	115.05 (17)	C28—C27—C26	119.9 (2)
N1—C14—H14A	108.5	C28—C27—H27	120.0
C15—C14—H14A	108.5	C26—C27—H27	120.0
N1—C14—H14B	108.5	C27—C28—C23	121.1 (2)
C15—C14—H14B	108.5	C27—C28—H28	119.4
H14A—C14—H14B	107.5	C23—C28—H28	119.4
O3—C15—C14	110.10 (17)		
			
O1—S1—N1—C14	−152.35 (16)	C7—C8—C13—C12	−177.9 (2)
O2—S1—N1—C14	−23.12 (19)	C11—C12—C13—C8	−0.5 (4)
C1—S1—N1—C14	92.00 (18)	C7—N1—C14—C15	66.9 (2)
O1—S1—N1—C7	34.24 (19)	S1—N1—C14—C15	−106.61 (19)
O2—S1—N1—C7	163.48 (15)	N1—C14—C15—O3	62.2 (2)
C1—S1—N1—C7	−81.40 (18)	N1—C14—C15—C16	−177.57 (18)
O1—S1—C1—C2	135.22 (18)	C17—N2—C16—C22	135.09 (19)
O2—S1—C1—C2	6.6 (2)	C17—N2—C16—C15	−100.6 (2)
N1—S1—C1—C2	−108.60 (19)	O3—C15—C16—N2	−170.49 (15)
O1—S1—C1—C6	−45.3 (2)	C14—C15—C16—N2	68.9 (2)
O2—S1—C1—C6	−173.95 (17)	O3—C15—C16—C22	−47.9 (2)
N1—S1—C1—C6	70.89 (19)	C14—C15—C16—C22	−168.55 (17)
C6—C1—C2—C3	−0.7 (3)	C16—N2—C17—O4	4.3 (3)
S1—C1—C2—C3	178.78 (18)	C16—N2—C17—O5	−176.89 (17)
C1—C2—C3—C4	−0.8 (4)	C18—O5—C17—O4	4.3 (3)
C2—C3—C4—F1	−178.3 (2)	C18—O5—C17—N2	−174.58 (17)
C2—C3—C4—C5	1.8 (4)	C17—O5—C18—C20	174.12 (18)
F1—C4—C5—C6	178.8 (2)	C17—O5—C18—C21	−67.5 (2)
C3—C4—C5—C6	−1.2 (4)	C17—O5—C18—C19	57.5 (2)
C4—C5—C6—C1	−0.3 (3)	N2—C16—C22—C23	−56.3 (2)
C2—C1—C6—C5	1.3 (3)	C15—C16—C22—C23	−178.44 (17)
S1—C1—C6—C5	−178.21 (18)	C16—C22—C23—C24	112.4 (2)
C14—N1—C7—C8	58.6 (3)	C16—C22—C23—C28	−67.9 (2)
S1—N1—C7—C8	−127.76 (17)	C28—C23—C24—C25	−0.5 (3)
N1—C7—C8—C13	−112.8 (2)	C22—C23—C24—C25	179.28 (19)
N1—C7—C8—C9	68.8 (3)	C23—C24—C25—C26	0.9 (3)
C13—C8—C9—C10	0.1 (3)	C24—C25—C26—C27	−0.4 (3)
C7—C8—C9—C10	178.6 (2)	C25—C26—C27—C28	−0.6 (4)
C8—C9—C10—C11	−0.9 (4)	C26—C27—C28—C23	1.1 (3)
C9—C10—C11—C12	0.9 (4)	C24—C23—C28—C27	−0.5 (3)
C10—C11—C12—C13	−0.2 (4)	C22—C23—C28—C27	179.71 (19)
C9—C8—C13—C12	0.6 (3)		

### Hydrogen-bond geometry (Å, º)

Cg1 is the centroid of the C23–C28 ring.

**Table:**

*D*—H···*A*	*D*—H	H···*A*	*D*···*A*	*D*—H···*A*
N2—H2n···O4^i^	0.88 (2)	2.36 (2)	3.179 (2)	154 (2)
O3—H3o···O2^i^	0.84 (2)	2.08 (2)	2.904 (2)	166 (3)
C7—H7*A*···O2^i^	0.99	2.56	3.405 (3)	143
C22—H22*A*···O4^i^	0.99	2.57	3.358 (2)	137
C20—H20*C*···*Cg*1^ii^	0.98	2.78	3.719 (2)	160

Symmetry codes: (i) *x*+1, *y*, *z*; (ii) *x*−1, *y*, *z*−1.
